# Genetic impact of copy number variations on congenital heart defects: Current insights and future directions

**DOI:** 10.1016/j.gmg.2024.100008

**Published:** 2024-11-22

**Authors:** Nandini Krishnamurthy, Devi Krishna, Sanjana, Jebaraj Rathinasamy, Ashok Kumar, Andrea Mary Francis

**Affiliations:** a Department of Human Genetics, Sri Ramachandra Institute of Higher Education and Research, Chennai, Tamil Nadu, India; b Department of Pediatric Cardiology, Sri Ramachandra Medical Centre, Chennai, Tamil Nadu, India; c Department of Biotechnology, Vels Institute of Sciences Technology and Advanced Studies (VISTAS), Chennai, Tamil Nadu, India

**Keywords:** Copy number variation, Genomic instability, Multiplex ligation-dependent probe amplification (MLPA), Chromosomal microarray analysis (CMA), Whole exome sequencing (WES), CNV-specific sequencing (CNV-seq), Short Interspersed nuclear element, Long interspersed nuclear element

## Abstract

Congenital heart defects (CHDs) are the most prevalent congenital abnormalities, and they are commonly associated with genetic alterations, namely copy number variants. CNVs, which are duplications or deletions of DNA sequences, can disrupt gene regulation, impact dosage-sensitive genes, and cause loss-of-function mutations, all of which can interfere with heart development. CNVs cause genomic instability by changing essential genes, which plays an important role in the pathophysiology of CHDs. Detecting these variants is critical for better understanding the genetic causes of these abnormalities and improving patient outcomes. Advanced genetic testing tools aid in detecting CNVs linked to CHDs. Multiplex Ligation-Dependent Probe Amplification (MLPA), High-throughput Ligation-Dependent Probe Amplification (HLPA), Whole Exome Sequencing (WES), Chromosomal Microarray Analysis (CMA), and CNV-specific sequencing (CNV-seq) have all greatly improved the detection of these variants. Furthermore, whole genome sequencing (WGS) has emerged as a potent method for detecting CNVs on a wide scale, allowing for earlier diagnosis and more effective treatment planning. Therefore, this review focuses on the rising significance of CNV research in congenital heart defects, emphasizing on how genetic differences might lead to improved diagnostic and treatment options. By combining genomic technologies, researchers and clinicians can gain a better understanding of the function of CNVs in CHDs, opening the door for personalised therapy.

## Introduction

Congenital heart defects (CHDs) are the most prevalent type of congenital abnormalities in humans and are a leading cause of morbidity and infant death worldwide [1]. Congenital heart disease (CHD) is a collection of congenital defects affecting the structure and function of the heart or blood vessels, even if the condition is discovered later in life [2]. Small lesions with no clinical symptoms to potentially deadly conditions are all included in this spectrum of heart abnormalities. Globally, there are 8 cases of CHD for every 1000 live births, and more recent research has shown that the number can rise as high as 9.5 cases [3]. Regional studies have revealed occurrences ranging from 1.2 to 17 per 1000 live births, despite the fact that different criteria make quantification challenging [4]. Many abnormalities are missed by regular prenatal ultrasound testing, despite the fact that fetal echocardiography can identify the majority of heart problems in utero [5]. Results can be improved if severe CHD is diagnosed in utero. The kind and degree of the cardiac abnormality influence the signs and symptoms [6]. Not all heart lesions are discovered in children, but majority are. A new cardiac phenotype with distinct late consequences is produced by surgical repair. CHD continues to be the primary cause of non-infectious newborn mortality worldwide, although advancements in early identification and surgical correction. Most people who survive this early stage need specialized cardiac care for the rest of their lives. Also, CHD's genetic foundations highlight the significance of genetic mutations and variations [7]. An estimated 40 % of instances of CHD have specific hereditary origins. Chromosome abnormalities, or aneuploidies, account for 13 % of the genetic causes of CHD (estimated; range: 9 % to 18 %) and copy number variations (CNVs) account for 10 % to 15 % of the genetic causes (estimated: 3 % to 25 % in syndromic CHD and 3 % to 10 % in non-syndromic CHD) [8], [9]. Moreover, population-level study has repeatedly shown that carriers of CNVs in particular genomic hotspots have a higher burden of CHD than controls, indicating the pathogenic potential of uncommon or de novo CNVs in the contribution to CHD [10], [11]. The concept of copy number variation, or CNV, describes an event where the number of copies of a certain DNA region differs across the genomes of different people. Each particular variation could have thousands of bases or only a few hundred. These structural variations, which can impact large regions of DNA, may have resulted from duplications, deletions, or other modifications. Genes may or may not be present in such locations [12].

## Background

Duplicate or deleted DNA segments (less than 50 bp), and are known as copy-number variations (CNVs), and they are a significant source of genetic diversity across individuals [13], [14]. CNVs are a very varied class of mutations that may cause disruption of regulatory sequences, gain or loss-of-function (LoF) through gene truncation or fusion, change the copy number of dosage sensitive genes, and unmask recessive alleles. As such, they remain as powerful phenotypic modifiers [15]. A few genomic regions (1q21.1, 2q13, 8p23.1, 11q24, 15q11.2, 16p11.2, and 22q11.2) include CNVs that are frequently abundant in CHD cohorts and impact dosage-sensitive transcriptional regulators necessary for the development of the heart [16]. At present, around 100 genomic disorders (GDs), or illnesses caused by rearrangements in the genome, are currently identified [17]. Some of these CNVs, which are adverse, have repetitions surrounding them and reoccur, appearing in the population at a low but steady frequency [18]. Large biobanks that link genotype to phenotype data have emerged, which has encouraged research on CNVs in the general population. The most effective method for describing the entire landscape of human CNVs is whole genome sequencing [19], [20]. On the other hand, exome sequencing data can be obtained with higher sample sizes, which provides an opportunity to evaluate the phenotypic impact of minor CNVs [21], [22]. According to a model of variable expressivity, which is consistent with findings for point mutations, CNVs can result in a broad range of phenotypic alterations, from mild subclinical symptoms to severe early-onset diseases. This raises the question of whether these loci are also linked to common diseases [23], [24].

## Mechanisms of copy number variation

**Fig. 1:**
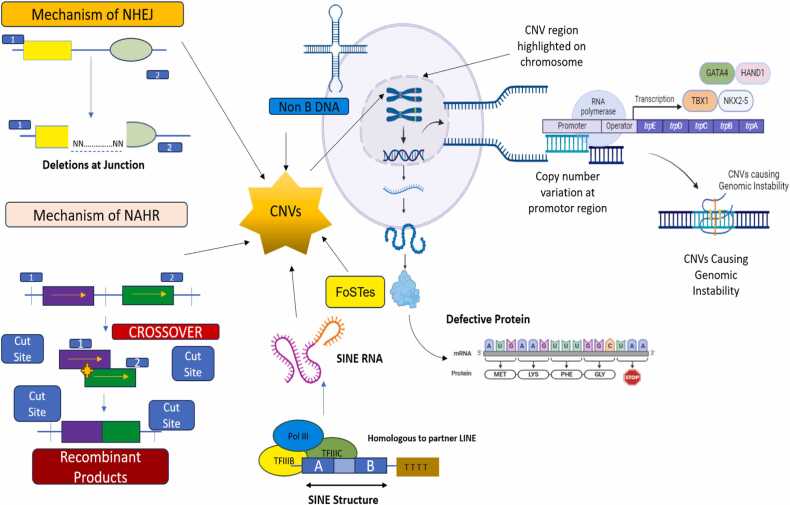
Mechanisms Leading to CNV Formation and Its Role in Genomic Instability and Congenital Heart Defect. The mutational mechanisms contributing to CNV (Copy Number Variation) formation, including recombination-based processes such as non-allelic homologous recombination (NAHR), non-homologous end joining (NHEJ) leads to genomic instability. Additionally, the replication-based mechanism fork stalling and template switching (FoSTeS) is depicted, explaining complex genome rearrangements. The enrichment of repetitive sequences, such as low-copy repeats (segmental duplications) and high-copy repeats (SINEs, LINEs) at CNV breakpoints contributes to genomic instability. Non-B DNA structures, like cruciforms formed by short repetitive motifs, also induce replication errors that further drive CNV formation.

**Fig. 2:**
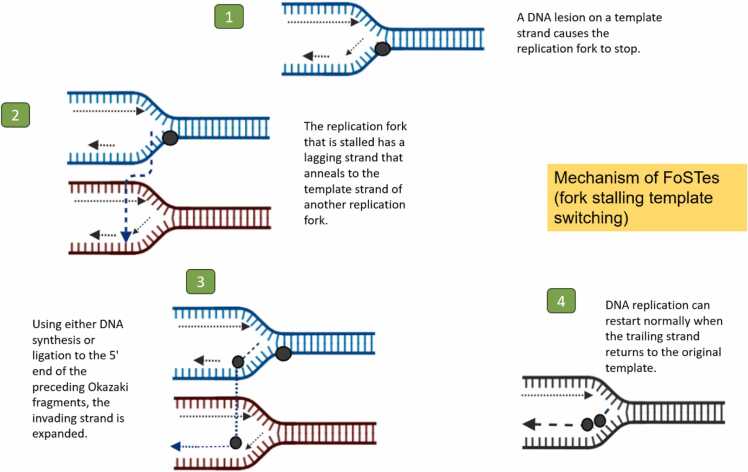
Mechanism by which FoSTes (fork stalling template switching) cause reorganizations in the genome.

Different mutational mechanisms, such as those linked to DNA recombination, replication, and repair, give rise to CNVs. Using the analysis of CNV breakpoint junction sequences, mechanisms of variation in gene copy number have been thoroughly explored. Since repeat sequences are enriched in the area of breakpoints, they play a significant role in CNV instability. These sequences include low-copy repeats like segmental duplications and high-copy repeats like SINEs, LINEs, and endogenous retroviruses [25]. Several mechanisms has been reported that a number of recombination based processes, such as retrotransposition, the activation and insertion of retrotransposons, non-allelic homologous recombination (NAHR) and non-homologous end joining (NHEJ) are responsible for the emergence of CNV (Fig. 1). To account for complex rearrangements not explained by the aforementioned mechanisms, a novel replication-based mechanism called fork stalling and template switching (FoSTeS) has been proposed (Fig. 2) [26]. Short repetitive sequence motifs, like inverted repeats, have the potential to adopt non-B DNA structures, like cruciforms, which can lead to replication errors and induces CNV [25]. Additionally, these sequences that do not form B-DNA are enriched in promoter regions. This also suggest that the same characteristics that permit regulation of transcription might also be mutagenic in the context of CNV formation. As a result, CNVs may have an impact on how gene regulation evolves [27].

## Copy number variation associated with CHDs

CNVs may interfere with the function of certain genes. Different CHDs can result from these variations, which can have a major impact on heart development. Deletions affecting the TBX1 gene in the 22q11.2 region are strongly linked to Tetralogy of Fallot (TOF) a complex defect involving pulmonary stenosis, right ventricular hypertrophy, aortic overhang, and ventricular septal defect (VSD) [25]. These duplications may affect the VNTR locus. Intron 4 of the NOS3 gene contains one of the well-studied VNTRs in connection to CHDs. The common alleles of this VNTR are 4a (four repeats) and 4b (five repeats). It is a 27 base pair repeat. Reduced NOS3 expression has been linked to the 4b allele, which may affect a person's vulnerability to CHDs [25]. SNPs in the NKX2–5 gene have been linked to outflow tract defects (tetralogy of Fallot, double-outlet right ventricle, and transposition of the great arteries) and septal defects (ASDs and VSDs) in about 2–4 % of all CHD cases. There are also reports of CNVs involving this gene where one patient with a triplication of the 5q35.3 region, which includes the NKX2–5 gene, was discovered via Multiplex Ligation-Dependent Probe Amplification (MLPA) screening of a multicentric CHD cohort. ASD, pulmonary stenosis, and extracardiac findings (brachydactyly, inguinal and umbilical hernias, hypospadias, microcephaly, and psychomotor delay) were all present in the patient [28], [29]. Strong links have been observed between overlapping 225–500 kb CNVs in 15q11.2 that impact four genes (TUBGCP5, CYFIP1, NIPA1, and NIPA2) and neurodevelopmental disorders like autism, schizophrenia, ADHD, and epilepsy. Additionally, CHD cohorts with LSL (OR 16, 95 % CI 2–120) and anomalous pulmonary venous connection (OR 14, 95 % CI 2–100) exhibit a significant enrichment of deletions in this CNV region [30], [31]. Carriers with CNV may have PDA as well as atrial or ventricular septal abnormalities. Similar to the nearby Prader-Willi/Angelman imprinted region, this CNV exhibits the parent of origin effect, in which deletions inherited from the mother are linked to neurodevelopmental disorders and deletions inherited from the father are linked to congestive heart failure [32], [33].

## Recurrent and “*de novo”* copy number variations in CHD

Tetralogy of Fallot (TOF, OR 22, 95 % CI 2.6–190) was the first condition in which recurrent de novo duplications of 1q21.1 were discovered however, they are also linked to numerous CHD abnormalities [34], [35]. Although considerably less frequent, deletions in this area are linked to a greater range of non-TOF CHD conditions, including complicated CHD lesions, septal abnormalities, and LSL [36]. There were recurrent 8p23.1 deletions and duplications found in the TOF, LSL, and single ventricle cohorts [37], [38]. A syndrome characterized by distinctive facial features, speech or developmental delay, and low penetrance congenital heart disease (25 %) is brought on by large duplications (∼3.6 Mb) involving GATA4 [39]. Notably, GATA4 duplications are often inherited from parents without congenital heart disease and are present in unselected control genotypes. On the other hand, 8p23.1 deletions are rare or absent in control groups and are linked to a variety of CHD conditions, including heterotaxy, Ebstein anomaly, AV canal abnormalities, left ventricular noncompaction, and hypoplastic left heart [38], [39]. Neurodevelopmental and CHD characteristics are also caused by recurrent and highly penetrant CNVs in 16p11.2. A 600 kb area of 16p11.2 that contains 29 genes has been linked to microdeletions and microduplications that has association to schizophrenia, autism spectrum disease, and alterations in brain size [40], [41]. TOF, LSL, septal abnormalities, and more complex lesions are among the various forms of congenital heart disease (CHD) that are substantially enriched in duplications or deletions that overlap with the VCFS area in 22q11.2. Due to non-allelic homologous recombination, eight low copy repeats (A–H) in 22q11.2 mediate frequent CNVs in this region [42]. Distal deletions or duplications of 22q11.2 (D-H) or reciprocal duplications of the common VCFS interval (A-D) can cause CHD with the absence of syndromic signs and symptoms [43]. In 30–60 % of cases, rare deletions in 2q13 that overlap with a 500 Kb interval result in intricate and variable CHD lesions, such as coarctation, septal defects, and AV canal defect [44]. Certain types of CHD lesions are linked to other recurrent CNVs. Sporadic cases of TOF without the distinguishable characteristics of Noonan syndrome are enriched in CNVs that overlap with the RAF1 gene in 3p25.2 [45]. In cohorts with LSL, transposition, and single ventricle physiology, recurrent large duplications of 16p13.1, involving the MYH11 gene, were discovered [46], [47]. Although biventricular repair is typically feasible, there have been isolated cases of patients with functional single ventricle cardiac disease and chromosome 22q copy number variants. Chromosome 22q copy number variants are associated with a rare form of congenital heart disease (CHD) that usually presents as hypoplastic left heart syndrome with a typical cytogenetic microdeletion. This condition often requires single ventricle reconstruction [48].

## Genetic testing for CNVs

Genetic testing for CNVs has emerged as an important method for both the diagnosis and characterization of CHDs. Copy number variant (CNV) genetic testing can be used for detecting and describing congenital heart defects (CHDs) since it can detect genetic anomalies associated with a variety of syndromes, including Turner and DiGeorge syndromes. We discuss a number of genetic testing techniques that allow for more detailed and accurate characterization of cardiac defects, with a higher diagnostic yield than conventional karyotyping. Furthermore, CNV testing makes early prenatal diagnosis possible, which allows for prompt interventions and improved planning for impacted infants. Additionally, it gives families vital information about the likelihood of recurrence and guides to personalized treatment plans, which eventually enhances therapeutic results and advances studies on the genetic causes of CHDs.

## Probe based techniques

### Multiplex ligation-dependent probe amplification (MLPA)

MLPA is a molecular method that identifies certain genomic regions, mainly exon-level CNVs. Pairs of oligonucleotide probes, which are short DNA molecules, designed to attach to certain genomic sequences are employed in MLPA to target specific regions of interest. By multiplexing several pairings, all of a gene's exons may be screened simultaneously. The ends of the probes ligate when they link together, but they only connect to their precise target sequence. PCR amplification can only take place if the probes have successfully ligated since each probe in the pair has a primer binding site. A method called capillary electrophoresis, which separates DNA fragments according to size, is then used to separate the resultant amplification products by length. The quantity of the target sequence contained in the sample directly correlates with the amount of amplified product produced by MLPA. Dosage anomalies, such as exon deletions or duplications, by comparing the variations in peak heights of these products from the sample to a reference sample with a known copy number can be detected [49]. A pilot study evaluated the use of MLPA to detect CNVs in children with congenital heart defects (CHDs). The study included 56 patients, half of whom had syndromic CHDs, and found that ventricular septal defect (VSD) was the most common heart defect. MLPA was used to investigate CNVs in the 22q11.2 region and genes such as GATA4, TBX5, NKX2–5, BMP4, and CRELD1. Two heterozygous deletions in the 22q11.2 region were identified in the syndromic group, while no CNVs were detected in the other genes. The study concluded that MLPA is a valuable first-line genetic test for syndromic CHDs, particularly for identifying CNVs in the 22q11.2 region, and recommended its inclusion in genetic screening panels for these patients [50]. MLPA and microarray analysis were used to evaluate copy number variations (CNVs) in congenital heart diseases (CHDs). Samples were collected from 18 prenatal cases, 16 isolated CHD cases, and 33 syndromic CHD cases. MLPA involved probe hybridization, ligation, amplification, and detection through capillary electrophoresis to determine the copy number of target sequences. Microarray analysis provided a genome-wide scan for CNVs. The combined approach of MLPA and microarray analysis enhanced the detection rate of CNVs, identifying pathogenic CNVs in 21 % of syndromic CHD cases, including deletions and duplications in regions associated with CHDs [51]. To detect copy number variations (CNVs) in Iranian families with non-syndromic congenital heart defects (CHDs), MLPA was used. It was revealed that MLPA is an effective method for early diagnosis of non-syndromic CHD patients, as it successfully identified CNVs in the 22q11.2 region in some patients which are associated with CHDs [52]. The genetic testing of newborns with critical congenital heart defects (CCHD) admitted to the intensive care unit (ICU), MLPA was used to detect CNVs. This study included 100 consecutive newborns with critical CHD, and MLPA was chosen for its rapid and cost-effective nature making it suitable for initial chromosomal analysis in a critical care setting. The findings revealed that MLPA identified pathogenic or likely pathogenic variants in 10 % of the newborns, with the most common variant being the deletion of the 22q11.2 region, associated with DiGeorge syndrome. For newborns with normal MLPA results, additional genetic testing using chromosomal microarray and clinical exome sequencing identified additional pathogenic variants in 3 % of the cases each, highlighting the importance of comprehensive genetic evaluation. The study also emphasized that early genetic diagnosis using MLPA can guide clinical management and decision making in newborns with critical CHD underscoring the utility of MLPA as a first-tier test, followed by more detailed analyses if initial results are normal. Also, MLPA is an effective and efficient method for initial genetic screening in newborns with critical CHD admitted to the ICU, providing valuable information for clinical management and further genetic counseling [53]. Hungarian pediatric and adult patients with CHDs were screened for CNVs in the 22q11.2 region using MLPA. The principle of MLPA remains the same as mentioned earlier [51]. Variations in probe quantity indicated the presence of CNVs, with reduced quantities suggesting deletions and increased quantities indicating duplications. In this study, MLPA was combined with chromosomal microarray analysis and droplet digital PCR to comprehensively assess CNVs, helping to identify rare pathogenic patterns in the 22q11.2 region among the patients [54].

## High-throughput ligation-dependent probe amplification (HLPA)

High-throughput ligation-dependent probe amplification is a modified method from MLPA. To determine how many copies of different genomic regions are present in a multiplex PCR reaction, the MLPA technique was modified to create HLPA. In order to be separated and measured using capillary electrophoresis a combination of amplification fragments of varying lengths and tagged fluorophore is created. Each probe has an adaptor sequence to allow amplification using universal primers and a target-specific sequence complementary to the genomic target just as MLPA. By using a ligation template and two lengthening ligation probes to further lengthen the downstream probe, HLPA introduces a lengthening ligation system that allows for the simultaneous sorting of numerous amplification products based on size. This is in contrast to MLPA, which uses a stuffer sequence in the downstream probe that can reach a length of 200 bp making it difficult to be chemically synthesized. Because of their complementary sequences to the ligation template, one lengthening probe can hybridize right next to another. Following hybridization, a thermostable ligase simultaneously ligates the ligation template and the genomic DNA specific probes. The single or double ligated probes are then amplified by the universal PCR primers, producing a continuous DNA fragment [55].

It is one of the emerging techniques to identify CNVs. A reliable method for CNV screening that has an appropriate cost-benefit ratio, a quick turnaround time, and high multiplex PCR system compatibility is the high-throughput ligation-dependent probe amplification (HLPA) assay [56]. The impact of chromosomal abnormalities on the surgical outcomes of Chinese pediatric patients with congenital heart disease (CHD) using high-throughput ligation-dependent probe amplification (HLPA) was done to detect copy number variations (CNVs) in 1762 children who underwent cardiac surgery. The study found that 21.45 % of the patients had at least one CNV, with 2.38 % carrying multiple CNVs, and the detection rate of pathogenic CNVs (ppCNVs) was 9.19 %, significantly higher than in healthy Han Chinese individuals. Patients with ppCNVs required more complex surgeries and had longer durations of cardiopulmonary bypass and aortic cross-clamp procedures. Additionally, the atrioventricular septal defect (AVSD) subgroup had a higher detection rate of ppCNVs compared to other CHD subgroups. The study concluded that CNVs are significant contributors to CHD in Chinese children and highlighted the effectiveness of HLPA in genetic screening for CNVs in CHD patients [57]. A study involved 32 Colombian patients with isolated congenital heart disease (CHD) during the neonatal period. Genomic DNA was isolated from peripheral blood samples collected from these patients. Using High-throughput Ligation-dependent Probe Amplification (HLPA), the study detected copy number variations (CNVs) in the 22q11.2 chromosomal region. HLPA successfully identified CNVs in 21.9 % of the patients, confirming its effectiveness in detecting CNVs associated with CHD [58].

## Sequencing based techniques

### Whole exome sequencing (WES)

Whole exome sequencing (WES) was utilized to identify copy number variations (CNVs) in congenital heart defects (CHDs) by sequencing the protein-coding regions of the genome. This approach allows for the detection of both small and large deletions or duplications within these regions, which may contribute to CHDs. By focusing on the exome, WES provides a detailed view of genetic variations that might not be captured by other methods, such as chromosomal microarray analysis (CMA), making it particularly valuable in identifying pathogenic CNVs associated with CHDs. Also, the study concluded that combining whole exome sequencing (WES) with CNV-seq significantly enhances the detection of genetic abnormalities in fetuses with congenital heart defects (CHDs). This integrated approach provides a more comprehensive genetic diagnosis, identifying both single nucleotide variants and copy number variations that might be missed by traditional methods. This improved diagnostic capability can lead to better-informed clinical decisions and management of CHDs in prenatal settings [59]. WES was used to identify rare damaging variants contributing to sex differences in congenital heart disease (CHD), including the detection of copy number variations (CNVs). CNVs were identified using algorithms that analyze the read-depth of exome sequencing data, comparing the number of reads mapped to each exon against a reference to pinpoint regions with abnormal copy numbers. These detected CNVs were further validated using chromosomal microarray analysis (CMA) to ensure accuracy. This study demonstrated the effectiveness of exome sequencing in providing a comprehensive genetic analysis of CHD by detecting both single-nucleotide variants (SNVs) and CNVs [60]. Exome sequencing is a valuable tool for detecting copy number variations (CNVs) associated with congenital heart defects (CHDs) in both singleton and twin fetuses. Exome sequencing could identify CNVs that were not detected by traditional methods, highlighting the important of WES in the genetic diagnosis of CNVs in CHD [61].

## Genome sequencing (GS) for genomic testing

Genome sequencing includes the majority of nuclear DNA, whereas exome sequencing concentrates on the ∼1–2 % of DNA that codes for proteins. This covers promoters and transcription enhancers that are not located within exons and may play a role in the pathophysiology of CHD. Additionally, CNVs, specific structural variations, and intronic variants not detectable by exome sequencing can be found using genome sequencing [62]. Genome sequencing has shown promise in treating individuals with cardiomyopathy and CHD, according to recent studies. In these trials, head-to-head comparisons showed that rapid genome sequencing excelled CMA in some cohorts of CHD patients, resulting in clinically actionable results in 27–46 % of individuals [63], [64]. Moreover, there are still gaps in the coverage of genome sequencing, even though it is better than exome sequencing [65]. Other studies, however, draw attention to specific drawbacks, such as the difficulty in identifying variants of indeterminate significance, with changes in variant interpretation up to 43 % [66].

## Copy number variation

### Sequencing (CNV-seq)

Blood from the fetal cord or amniotic fluid was used to obtain genomic DNA (gDNA). Ten and fifty nanograms of DNA were broken up, and libraries were created by end repair, ligation with sequencing adaptors, and polymerase chain reaction (PCR) amplification. Then, the NextSeq 500 platform (Illumina, San Diego, USA) was used to perform massively parallel sequencing on the DNA libraries, producing roughly five million raw sequencing reads that were 36 base pair long genomic DNA sequences. The Burrowse Wheeler algorithm was used to precisely and uniquely map 2.8–3.2 million reads, utilizing the hg19 genomic sequence as a reference [67]. The mapped reads from the p to q arms of the 24 chromosomes were gradually assigned to 20-kilobase (kb) bin sizes. The results were interpreted as increases and losses of copy number using a number of public databases, including the Database of Genomic Variants (DGV), Online Mendelian Inheritance in Man (OMIM), DECIPHER, University of California, etc. In accordance with the standards provided by the American College of Medical Genetics (ACMG), CNVs were evaluated and classified into five categories: pathogenic (P), likely pathogenic (LP), VOUS, likely benign (LB), and benign (B) [68].

## Microarray based techniques

### Chromosomal microarray analysis (CMA)

CMA was performed on 642 fetuses diagnosed with congenital heart defects (CHDs), enrolled from a single center over a six-year period (2017–2022). Both conventional karyotyping and chromosomal microarray analysis (CMA) were performed simultaneously on these fetuses. The study found that CMA had a diagnostic yield of 15.3 %, significantly higher than that of karyotyping. The diagnostic yields were particularly high in subgroups with complex CHD, conotruncal defects, right ventricular outflow tract obstructive defects (RVOTO), atrioventricular septal defects (AVSD), and left ventricular outflow tract obstructive defects (LVOTO). The study concluded that CMA is a reliable and high-resolution technique that should be recommended as the front-line test for prenatal diagnosis of fetuses with CHD. It highlighted the importance of CMA in detecting clinically significant chromosomal abnormalities, especially in non-isolated CHD cases with additional structural anomalies or soft markers, thereby underscoring its effectiveness in identifying CNVs associated with CHDs [69]. CMA was used to analyze 427 fetuses with CHDs, revealing a significant correlation between certain cardiac phenotypes and the presence of CNVs. CHDs combined with extracardiac abnormalities (ECAs) had a higher detection rate of chromosomal abnormalities, particularly in cases involving conotruncal defects and other specific cardiac phenotypes. This highlights the ability of CMA to uncover genetic factors that may influence the severity and complexity of CHDs. These findings underscore the necessity of CMA in prenatal diagnostics, as it enhances the detection of clinically significant chromosomal abnormalities, aiding in more accurate genetic counseling and prenatal diagnosis. By identifying specific CNVs and their associations with different CHD phenotypes, CMA provides valuable insights into the genetic underpinnings of CHDs, which can inform clinical management and decision-making for affected pregnancies. This makes CMA an essential tool in the genetic evaluation of CHDs, improving outcomes through early and precise detection of genetic anomalies [70]. CMA generally yields the following four results: (1) no abnormality (normal array); (2) pathogenic variant; (3) VUS; and, in rare cases, (4) secondary or incidental finding (Table 1). Groups 2–4 might co-occur. The American College of Medical Genetics and Genomics (ACMG) guidelines are widely adhered to laboratories when it comes to the interpretation and reporting of postnatal constitutional CNVs [71].

**Table 1:** Outcomes of CMA-based clinical genetic testing.

S. No	Outcome	Description	Example	Key Insights
1	Normal Array	No abnormality detected. The cause of the individual’s CHD remains unexplained.	A patient with truncus arteriosus and a loss-of-function mutation in NKX2 −5.	A negative result is the most common outcome. Further genetic testing may help explain CHD in the future. CMA does not detect small mutations or imbalances.
2	Pathogenic Variant	Pathogenic CNV known to increase risk for CHD. Referral to medical genetics is recommended. Parental testing may reveal recurrence risks.	A typical 1q21.1 duplication in a patient with Tetralogy of Fallot (TOF)[72].	Parental testing may identify CNVs or chromosomal rearrangements, increasing recurrence risk for future offspring.
3	VUS (Variant of Uncertain Significance)	CNVs that are not fully characterized as benign or pathogenic. Subcategories: ‘VUS; likely pathogenic’, ‘VUS; likely benign’, and ‘VUS (no sub-classification)’.	A duplication overlapping CACNA1C and a typical 15q11.2 deletion in a patient with Transposition of the Great Arteries (TGA)[73].	Genetic counseling is advised. Some VUS, especially 'likely pathogenic', may be reclassified as pathogenic over time with more research.
4	Secondary Finding	Variant associated with a genetic disorder unrelated to the primary referral reason, often linked to an adult-onset condition.	Haploinsufficiency of the BRCA2 gene in a patient with Tetralogy of Fallot (TOF)[74].	Rare, but may involve discovering a genetic condition unrelated to CHD, possibly associated with adult-onset diseases.

## Genetic testing panels

The introduction of DNA sequencing technology in 1977 marked a significant advancement in the field of genetic testing. This breakthrough made it possible to identify alterations in a single nucleotide [75]. Since then, targeted sequencing has been commercially accessible to discover single nucleotide genetic changes in certain genes or entire panels of genes linked to cardiomyopathies and congestive heart failure. This is because sequencing technology have advanced. Broader genomic testing could miss variations that are detected by targeted genetic testing, which offers more sequencing coverage for the target gene. It is crucial to understand the limits before performing or evaluating gene panel testing. For instance, whereas the majority of contemporary genetic testing panels combine deletion/duplication analysis and sequencing, these aspects are absent from many older modalities, which results in the missed identification of these variants. For instance, a frequent variation in MYBPC3 that can result in cardiomyopathy may be undetected by certain genetic panels. Panels that solely sequence exonic areas may not cover this variant because it is found in deep intronic regions [76].

## Impact of genetic testing methodologies on clinical outcomes in context to CHD

Recent developments in genomic technologies, including MLPA, HLPA, WES, GS and CMA, have greatly improved our capacity to identify and comprehend congenital heart disease (CHD) at the molecular level. Clinical decision-making and patient outcomes are impacted by the distinct insights that each of these methods offers into the genetic basis of CHD. The clinical impact of these approaches is summarized in the following Table 2, which also shows how they assisted in ensuring patients with CHD receive more individualized treatment, pregnancy decision, earlier interventions, and increased diagnostic accuracy.

**Table 2:** Clinical Implications and Patient outcomes of genetic testing for CNV detection in CHD.

Testing Technique	Description	Clinical Implications/Patient Outcomes
**MLPA**	To detect pathogenic CNVs in prenatal samples. Prenatal Screening of 33 fetus (32 CHD and 1 with Kidney defect).	Pathogenic CNVs were found in 5 samples (15.2 % of cases), enabling early identification of genetic causes of CHD[77].
	To identify microdeletions linked to DiGeorge syndrome.	Detected microdeletions in the 22q11.2 region (≈2 Mb) in two fetuses, including one distal microdeletion (≈416 kb) containing genes *LZTR1, CRKL, AIFM3*, and *SNAP29* in the fetus with bilateral renal agenesis and CHD[77].
	To detect microdeletions in regions associated with other syndromes (e.g., 8p23.1, 9q34.3).	Identified a 3.8 Mb microdeletion associated with 8p23.1 microdeletion syndrome and a 1.7 Mb microdeletion in region 9q34.3 related to Kleefstra syndrome[77].
	To screen recurrent CNVs in genomic regions (*GATA4, NKX2 −5, TBX5, BMP4, CRELD1*) and the 22q11.2 region in non-syndromic patients with cardiac septal defects**.**	CNVs were identified in 6.66 % of patients (3/45), along with three 22q11 deletions. This enabled early diagnosis of genetic syndromes associated with congenital heart defects (CHD) such as DiGeorge syndrome[78].
	To detect genetic abnormalities, including microdeletions and duplications in patients with CHD and extracardiac manifestations.	Identified abnormalities in 12.5 % of patient, resulted in diagnosing syndromes such as Williams-Beuren, DiGeorge (22q11.2 microdeletion), Wolf-Hirschhorn, and 2p25.3 microduplication with CHD manifestations[79].
	To detect CNVs in newborns with critical CHD using SALSA MLPA P250-B2 Di George and P311-B1 CHD kits.	Pathogenic CNVs were found in 10 % of patients (10/100). Nine patients had 22q11.2 deletion resulting in DiGeorge syndrome and one patient had a 3p25 deletion associated with CHD[80].
**HLPA**	To screen CNVs in Chinese children with CHD undergoing cardiac surgery, focusing on CNV regions with disease-causing potential.	Patients with atrioventricular septal defects (AVSD) showed a higher detection rate of ppCNVs (23.10 %), underscoring the importance of CNV screening using HLPA for surgical planning and patient outcomes[57].
	To detect chromosomal CNVs, specifically at the 22q11.2 locus, in patients with congenital heart disease (CHD).	Identified 22q11.2 deletions (39 cases) and duplications (1 case) in a large cohort (818 patients), enabling targeted genetic analysis. This allows for early intervention, personalized management, and informed reproductive counseling for families at risk[81].
**WES**	To identify pathogenic variations in TOF-related genes.	Detected mutations in 14 out of 17 TOF patients, enabling early diagnosis and better clinical management[82].
	To identify causative gene variants in a Chinese family with CHD.	Identified 4 mutation sites in the family. Two variants, c.3245 A>G (His1082Arg) of the *AMER1 gene* and c .238 G>C (Val80Leu) of the *KCNE1 gene*, were determined to be causative agents for CHD. The variant *AMER1* with X-linked recessive inheritance and *KCNE1* with autosomal dominant inheritance were linked to CHD development in this family. This helps the family with guidance on the inheritance risks for future generations and enables targeted advice for family planning[83].
	To identify rare, pathogenic variants segregating with CHD in familial cases.	Identified likely pathogenic mutations in 3 out of 9 families especially mutations *in GATA4, TLL1,* and *MYH11* genes contributed to CHD phenotypes followed by In-Silico analysis to predict the pathogenicity of identified variants. This enabled early identification of causative mutations in 33 % of families facilitating timely genetic counseling and targeted interventions for at-risk family members[84].
	To identify rare predicted damaging variants in 1726 candidate genes for laterality defects.	Identified 26 rare damaging variants and 2 hemizygous deletions, allowing for targeted diagnosis and timely interventions in 7.1 % of cases[85].
	To identify unique, deleterious genetic variants in 829 patients with non-syndromic TOF.	Identified variants in *NOTCH1* (4.5 %) and FLT4 (2.4 %) as major contributors to TOF. Enables genetic screening and early intervention[86].
**GS**	To investigate the utility of GS for prenatal diagnosis of CHD in fetuses with non-diagnostic karyotyping or chromosomal microarray results.	Provided a diagnosis for 4/13 (30.8 %) fetuses with complex CHDs, identifying pathogenic variants in genes such as *DNAH5, COL4A1, PTPN11* and *KRAS.* This aided in timely medical decision-making and counseling for parents regarding pregnancy continuation and potential interventions[87].
	To assess the genetic outcomes of a heterogeneous cohort of CHD patients.	GS identified clinically relevant genetic variants in known and emerging CHD genes. Through high-confidence gene screen (hcCHD) found actionable variants in 22 % of families, and a comprehensive analysis found additional actionable variants in 9 % of families. Overall, clinically relevant variants were identified for 31 % of families aiding in timely intervention and surgical planning for patients[88].
**CNV-seq**	To evaluate chromosomal aberrations in prenatal fetal diagnosis, focusing on copy number variations.	Detected abnormalities in 128 cases (19.48 %) out of 657. Identified 87 cases of CNVs in 78 samples, including 13 (14.94 %) that explicitly caused disease and 7 (8.05 %) that might cause disease. Demonstrated high sensitivity for identifying microdeletions and microduplications across all chromosomes[67].
	To serve as an alternative diagnostic method for detecting chromosomal anomalies in fetuses diagnosed with CHD.	Demonstrated 100 % diagnostic concordance with CMA in detecting all 21 pathogenic chromosomal abnormalities associated with CHD. Supported the reliability and accuracy of CNV-Seq as a prenatal technique[89].
**CMA**	To confirm and further analyze findings from MLPA in children with CHD.	High-resolution analysis confirmed CNVs and ensured accurate diagnosis, aiding in personalized care management for affected pregnancies[77].
	To analyze chromosomal abnormalities in patients with CHD who had normal MLPA results.	Detected a 2.27 Mb deletion in chromosome 2q22, including the *ZEB2* gene, leading to the diagnosis of Mowat Wilson syndrome in one patient[79].
	To identify pathogenic chromosomal abnormalities and submicroscopic copy number variations (CNVs) in fetuses with congenital heart disease (CHD).	Pathogenic chromosomal anomalies were found in 21 out of 115 (18.3 %) CHD fetuses. For isolated CHD, CMA detected two cases of DiGeorge syndrome and one case of 1q21.1 microdeletion, 16p11.2 microdeletion, Angelman/Prader Willi syndromes, and 22q11.21 microduplication syndrome. In CHD fetuses with additional structural abnormalities, CMA identified eight whole or partial trisomies (19.0 %) and five CNVs (11.9 %) associated with various syndromes[89].

## Future directions

Collectively, these individually uncommon CNVs gains and losses, inherited and “de novo” are significant genetic variables that lead to aberrant development of the human heart and large vessels. Variable cardiac and extra-cardiac expression to varying degrees characterizes all uncommon variations that have been sufficiently examined to date. A lot of them also show decreased penetrance. Reproductive fitness effects and ascertainment bias can make estimating these characteristics difficult [90], [91], [92], [93]. In human CHD, epistatic interactions are not fully understood. Our comprehension of expression modifiers will decide our capacity to offer personalized medicine and counseling. The levels of complexity that we might anticipate to face even in people with fatal single-gene mutations are elegantly demonstrated in research performed on mice with heterozygous Nkx2–5 mutations [94]. Defining the breakpoints carefully and examining the intact allele, for instance, would be the first step towards many CNVs. TAR syndrome caused by distal 1q21.1 deletions is the best example of this approach's effectiveness [95]. The contribution of uncommon sequence-based alterations to the genetic architecture of CHD is being encouraged to be taken into consideration in a manner similar to that of CNV and structural variation. The Center of Excellence in Genomic Medicine Research have improved clinical diagnostic yield and advanced our knowledge of the aetiopathogenesis of CHD. Recently, the finding of new genes has started to be facilitated by whole-exome sequencing [96].

Further research on CNVs and CHD should focus on improving our knowledge of genotype-phenotype relationships. Expanding the use of whole genome sequencing (WGS) and high-resolution chromosomal microarrays may enable more complete identification of CNVs, including smaller, submicroscopic variations that may contribute to CHD risk [19], [20]. This will allow for the discovery of novel CNVs that have not previously been associated to CHD, as well as deeper characterization of recognised hotspots for CNV activity, such as 1q21.1, 22q11.2, and others [16]. Furthermore, improving CNV detection methods in prenatal settings, such as adding CNV analysis into regular fetal echocardiography, can help with CHD early identification and intervention efforts [5]. As huge biobanks expand, additional possibilities will arise to investigate the population-level impact of CNVs on heart development and the related risk factors for CHD. These databases should include varied populations to guarantee that CNVs and their consequences are thoroughly explored across ethnic and genetic backgrounds [17]. Furthermore, future study might investigate the function of CNVs in impacting the long-term outcomes of CHD cases that have been surgically fixed, particularly in finding CNVs that may predispose individuals to later-life cardiovascular difficulties or sequelae from the original abnormalities [10], [11]. Functional investigations should seek to understand the exact biological processes by which CNVs impair gene function or regulatory networks, particularly dosage-sensitive genes implicated in heart development [15]. This might lead to the development of gene therapy or pharmacological treatments for CNV-induced defects. The interaction of CNVs with other genetic abnormalities or environmental variables in regulating CHD risk will be a significant field of investigation, especially with the emergence of precision medicine techniques [23], [24].

Also, the function of oligogenic inheritance, epigenetic modification, genetic mosaicism, and noncoding variations in regulating the expression of potential CHD associated genes is being uncovered by next generation sequencing. Clinical risk prediction using these parameters is still difficult though. Thus, research using “single-cell sequencing” and “human-induced pluripotent stem cells” aids in developing preclinical frameworks for assessing the importance of novel genetic variations [97]. Advancement in sequencing technology has led to long read sequence (LRS) technology for structural variants. DNA segments longer than 10,000 bp can be sequenced using LRS. The two main LRS technologies at the moment, Oxford Nanopore Technology and Pacific Biosciences, are founded on distinct methodologies. The first is based on real-time sequencing of single molecules and uses a DNA polymerase to identify nucleotide incorporation events in real-time with high accuracy up to 99.8 % with circular consensus sequencing [98]. Emerging technologies like CRISPR based genomic approach, AI based machine learning model (AI/ML) for CNV prediction and exploring mitochondrial DNA variants could deepen our understanding for CNVs in CHD. For genetic findings to be responsibly translated into clinical practice, ethical and social issues must also be addressed. Finally, when data from whole genome and exome sequencing become available, combining CNV analysis with other omics methods, like as transcriptomics and proteomics, may give a more complete knowledge of the biochemical pathways affected in CHD. This integrated strategy may aid in the development of more personalised treatment strategies, resulting in better patient outcomes and perhaps lowering the lifelong burden of CHD.

## Abbreviations

CNV, Copy Number Variation; SINE, Short Interspersed Nuclear Element; LINE, Long Interspersed Nuclear Element; NAHR, Non-Allelic Homologous Recombination; NHEJ, Non-Homologous End Joining; FoSTeS, Fork Stalling and Template Switching; VNTR, Variable Number of Tandem Repeats; NOS3, Nitric Oxide Synthase 3; MLPA, Multiplex Ligation-Dependent Probe Amplification; CMA, Chromosomal Microarray Analysis; HLPA, High-throughput Ligation-Dependent Probe Amplification; WES, Whole Exome Sequencing; CCHD, Critical Congenital Heart Defect; ppCNV, Pathogenic Copy Number Variant; VCFS, Velocardiofacial Syndrome.

## Declaration of Competing Interest

Authors does not have any conflict of interest.
